# Shared Physiologic Pathways Among Comorbidities for Adults With Cerebral Palsy

**DOI:** 10.3389/fneur.2021.742179

**Published:** 2021-10-04

**Authors:** Daniel G. Whitney, Mary Schmidt, Edward A. Hurvitz

**Affiliations:** ^1^Department of Physical Medicine and Rehabilitation, University of Michigan, Ann Arbor, MI, United States; ^2^Institute for Healthcare Policy and Innovation, University of Michigan, Ann Arbor, MI, United States

**Keywords:** cerebral palsy, comorbidities, mortality, multimorbidity, clinical epidemiology

## Abstract

**Objective:** Aging with cerebral palsy is accompanied by a declining health and function status across neurological and non-neurological systems. There is a need to understand the shared pathophysiology among comorbidities for adults with cerebral palsy, to inform clinical assessment and guidelines for interventions to improve healthful aging. To begin defining multimorbidity, this study identified the most common comorbidity combinations and their association with mortality among a representative sample of adults with cerebral palsy.

**Methods:** Data from 2016 to 2018 were used from a random 20% sample from the fee-for-service Medicare database. Adults ≥18 years with cerebral palsy and 25 neurological and non-neurological comorbidities were obtained from 2016. Principal component (PC) analysis identified the most common comorbidity combinations, defined as individual PCs. Cox regression estimated the hazard ratio (HR) of 2-year mortality including all PCs and demographics in a single model. To facilitate comparisons, PC scores were transformed into quintiles (reference: lowest quintile).

**Results:** Among the 16,728 adults with cerebral palsy, the most common comorbidity combinations (PCs) in order were: cardiorespiratory diseases, dysphagia, and fluid/electrolyte disorders; metabolic disorders (e.g., diabetes, renal disease, hypertension); neurologic-related disorders (e.g., dementia, cerebrovascular disease); gastrointestinal issues; and orthopedic-related disorders. During the 2-year follow-up, 1,486 (8.9%) died. In the adjusted model, most PCs were associated with an elevated mortality rate, especially the first PC (5th quintile HR = 3.91; 95%CI = 3.29–4.65).

**Discussion:** This study identified the most common comorbidity combinations for adults with cerebral palsy, many of them were deadly, which may inform on the underlying pathophysiology or shared characteristics of multimorbidity for this population.

## Introduction

Neurological, functional, metabolic, and psychosocial aspects can manifest in multiple ways for children with cerebral palsy, and collectively increase risk for a variety of morbidities as these children become adults. The declining health and function with age can further complicate neurologic status in cerebral palsy (1–7), underscoring the importance of increasing knowledge of multimorbidity regarding the shared pathophysiology among neurological and non-neurological morbidities (8, 9).

Multimorbidity, defined as ≥2 morbidities, is a major burden for adults with cerebral palsy, and contributes to their greater healthcare utilization and costs (10). For example, 1 out of 4 adults with cerebral palsy aged 18–30 years have multimorbidity, which is >3 times more prevalent than the general population of young adults (11), and continues to get worse with older age (12). Recent work suggests that multimorbidity for adults with cerebral palsy is a robust risk factor for premature mortality (13, 14).

Importantly, many morbidities can be prevented, delayed, or better clinically managed; yet, the field lacks evidence-based approaches to monitor morbidities for adults with cerebral palsy. To address this need, the Whitney Comorbidity Index (WCI) was recently developed (15) and validated (14), which is a simple, clinical-friendly tool designed to monitor health status for adults with cerebral palsy. The WCI score is calculated by summing the presence of 27 morbidities across neurological and non-neurological systems, which captures the unique multimorbidity profiles for adults with cerebral palsy better than other currently available methods (15). However, while the WCI provides a novel basis to inform clinical decision making, it is a quantitative approach to defining multimorbidity profiles, and provides no evidence on the shared pathophysiology among comorbidities.

As the field progresses, there is a need to understand the shared etiologies and characteristics among groups of comorbidities for adults with cerebral palsy, to ultimately inform clinical assessment and guidelines for interventions. For example, a WCI of 3 identifies those at risk for premature mortality (14). However, if one person's multimorbidity is defined by hypertension, diabetes, and renal disease (WCI = 3), which are commonly comorbid in the general population (16), treatment strategies would be very different than someone with a multimorbidity defined by dysphagia, pneumonia, and mal-nutrition (WCI = 3). Given the difference in physiologic development for individuals with cerebral palsy, there may be unique combinations of comorbidities that may inform on the underlying shared pathophysiology across neurological and non-neurological systems.

Principal component analysis (PCA), a data reduction technique, is an excellent analytic approach to identify comorbidity combinations as it explains the variance within a dataset based on inter-correlations among variables. In the context of multimorbidity, PCA allows for meaningful analysis and clinically-relevant interpretation of the most common combinations of comorbidities. Accordingly, using a large, nationwide sample, the primary objective of this study was to perform PCA to identify the most common comorbidity combinations from the WCI present in adults with cerebral palsy. The secondary objective was to determine how each of these comorbidity combinations associated with mortality.

## Method

### Data Source

Data for this retrospective cohort study came from a random 20% sample of the Medicare fee-for-service claims database. Demographics and the WCI comorbidities were obtained from the year 2016. Death date was obtained from the years 2017 and 2018. As described previously (15), Medicare is a U.S. federal program providing health insurance coverage to adults ≥65 years or individuals across any age that have end-stage renal disease or one or more chronic disabilities, including cerebral palsy. While administrative claims data are primarily used for healthcare billing reimbursement, medical conditions can be identified by searching for unique International Classification of Diseases, Tenth Revision, Clinical Modification (ICD-10-CM) codes that are attached to individual claims, allowing for the possibility of health-related research. The ICD-10-CM codes used to identify cerebral palsy and WCI comorbidities have been presented elsewhere (14).

### Study Timeline and Sample Selection

The start date of follow-up was January 1, 2017 for all participants, which allows for a 1-year baseline period (17) and 2-years of follow-up for mortality. A flow diagram of the inclusion/exclusion criteria has been presented previously (14). Individuals that were 18 years of age or older at baseline with cerebral palsy were identified using an algorithm that required meeting at least one of the following criteria: one or more inpatient claims in 2016 for cerebral palsy; two or more outpatient claims in 2016 for cerebral palsy. Further inclusion criteria included: continuous enrollment in Part A and B from January 1, 2016 to January 30, 2017 (1 year + 30 days) to obtain a full 1-year baseline period and ≥30 days of follow up for mortality; complete data on demographics, including age, sex, race, and U.S. region of residence.

As epilepsy and intellectual disabilities often co-occur with cerebral palsy and can complicate the individual's medical needs (18, 19), a subgroup analysis was performed. For the subgroups, epilepsy and intellectual disabilities were identified in the same manner as cerebral palsy, and mutually exclusive groups were created: cerebral palsy only (CP only), cerebral palsy and co-occurring epilepsy but without co-occurring intellectual disabilities (CP+EP), cerebral palsy and co-occurring intellectual disabilities but without co-occurring epilepsy (CP+ID), and cerebral palsy and co-occurring epilepsy and intellectual disabilities (CP+EP+ID).

### Mortality

All-cause mortality up to December 31, 2018 (2-year period) was derived from the Medicare database. Medicare has >99% of deaths validated (20).

### WCI Comorbidities

The WCI comorbidities were previously identified using an iterative process that harmonized *a priori* clinical knowledge of relevant comorbidities for adults with cerebral palsy, use of established and validated measures, and data-driven approaches to select the final 27 WCI comorbidities (15). For the current study, two sets of two comorbidities were merged into a single comorbidity given the potential for considerable overlap, which may alter PCA interpretation: “any cancer” included the presence of “metastatic cancer” or “any malignancy, including lymphoma and leukemia, except malignant neoplasm of skin”; “type 2 diabetes” included the presence of “diabetes with chronic complication” or “diabetes without chronic complication” (*n* = 25 WCI comorbidities). Since the WCI includes epilepsy and intellectual disabilities as individual comorbidities, these were removed for the subgroup analysis, thereby totaling 23 comorbidities. All comorbidities were identified in the same manner as cerebral palsy.

### Statistical Analysis

PCA is a multivariable data reduction technique that operates in a highly correlated environment to identify inter-correlated variables. In the context of multimorbidity, PCA allows for meaningful analysis and interpretation of comorbidity data by reducing the number of comorbidities into a few inter-correlated combinations. Each comorbidity combination corresponds to a specific principal component (PC) (21, 22), which is independent of other PCs. To derive the PCs, the models included each of the WCI comorbidities (25 for the entire group; 23 for the subgroups). The PCA models used a varimax rotation to facilitate interpretation of the loading factors. Loading factors are derived from the correlation matrix and provides a numerical interpretation of the PCs. Comorbidities with a loading factor of ≥|0.40| were included for interpretation, which has been suggested previously (23). PCs with eigenvalues of ≥1.00 were retained and analyzed, as this is common practice for PCA (24).

The resulting PCs for the group analysis provides information on the most common comorbidity combinations. This group-level analysis also provides information on how much of the total variance of the data is explained for each PC, and when more than 1 PC, the cumulative variance explained. In this context, the explained variance provides indirect evidence of the heterogeneity of comorbidity profiles for adults with cerebral palsy, where a low cumulative percent for all identified PCs is indicative of a greater number of ways in which comorbidities are inter-related. PCA also provides individual-level data in a standardized manner, where each person is assigned a PC score for each PC separately with a mean of 0 and standard deviation of 1. For example, if a PC is defined by 4 comorbidities, individuals with 3 or 4 of those comorbidities would have a higher PC score compared to those with 2 or fewer.

Cox proportional hazards regression was used to determine the association between each of the identified PCs with mortality. Models were developed to estimate the hazard ratio [HR with 95% confidence intervals (CI)] of 2-year mortality after including all PCs in a single model, while controlling for age, sex, race, and U.S. region of residence. To facilitate comparisons across PCs and enhance clinical interpretation, individual-level PC scores were transformed into quintiles and the lowest quintile (lower likelihood of having the PC's comorbidities) was set as the reference. Interpretation of the HR is the risk of mortality per increase in a comorbidity profile that is more exemplary of that PC.

Analyses were performed using SAS version 9.4 (SAS Institute, Cary, NC, USA) and *P* ≤ 0.05 was considered statistically significant.

## Results

Baseline descriptive characteristics and prevalence of the WCI comorbidities for the entire group of adults with cerebral palsy (*n* = 16,728) and the subgroups, CP only (*n* = 7,542), CP+EP (*n* = 2,607), CP+ID (*n* = 2,781), and CP+EP+ID (*n* = 3,798), is presented in Table 1.

**Table 1:** Baseline descriptive characteristics and prevalence of comorbidities for the entire group of adults with cerebral palsy (CP) and for mutually exclusive subgroups based on co-occurring epilepsy (EP) and intellectual disabilities (ID).

	**Entire group** **(*n* = 16,728) (%)**	**CP only** **(*n* = 7,542) (%)**	**CP+EP** **(*n* = 2,607) (%)**	**CP+ID** **(*n* = 2,781) (%)**	**CP+EP+ID** **(*n* = 3,798) (%)**
**Descriptive characteristics**					
Age, mean (SD)	51.0 (15.3)	53.0 (16.2)	47.2 (15.2)	52.2 (14.3)	49.0 (13.3)
18–40 years	27.7	25.2	38.8	23.2	28.3
41–64 years	51.6	48.1	45.5	56.4	59.2
≥65 years	20.7	26.7	15.7	20.4	12.5
Sex					
Women	48.2	51.2	52.1	51.1	53.3
Men	51.8	48.9	47.9	48.9	46.7
Race					
White	80.5	80.7	77.6	82.0	81.2
Black	13.0	13	13.6	13.1	12.7
Hispanic	3.3	1.1	1.4	1.4	1.3
Asian	1.0	1.0	1.4	0.8	0.8
North American Native	0.8	3.3	4.6	2.3	3.3
Other	1.2	0.9	1.4	0.5	0.7
U.S. region of residence					
Midwest	27.7	27.5	26.0	29.1	28.4
Northeast	21.7	19.2	16.4	27.6	26.0
South	34.4	36.0	39.0	29.9	31.4
West	16.2	17.3	18.6	13.5	14.2
**Comorbidities**					
Hypertension (Un)complicated	45.0	51.3	39.6	43.6	37.3
Intellectual disabilities	39.3	-	-	-	-
Epilepsy	38.3	-	-	-	-
Other neurological disorders	35.8	15.4	54.6	26.8	69.9
Depression	28.2	30.2	27.3	31.1	22.8
Fluid and electrolyte disorders	25.5	19.7	24.6	23.4	39.0
Gastrointestinal issues	23.8	19.3	20.9	28.1	31.7
Dysphagia	20.9	13.5	18.1	24.3	34.8
Hypothyroidism	20.7	16.5	17.7	22.3	30.0
Bone fragility	20.7	16.1	18.2	21.6	31.1
Osteoarthritis and allied disorders	19.6	23.7	17.2	16.9	15.1
Cardiac arrhythmias	18.5	15.8	18.1	18.7	24.1
Type 2 diabetes	17.6	19.7	14.8	18.5	14.9
Pneumonia	14.7	9.2	13.7	15.4	25.8
Chronic pulmonary disease	14.0	14.0	14.6	12.5	14.5
Blood loss and deficiency anemias	12.7	11.2	11.9	13.7	15.4
Cerebrovascular disease	9.8	9.0	13.9	6.2	11.1
Congestive heart failure	8.3	8.5	6.9	8.4	8.9
Neurogenic bowel or bladder	7.5	8.1	7.3	6.7	7.3
Renal disease	7.1	8.0	6.7	6.0	6.4
Dementia	6.4	5.0	6.3	7.5	8.5
Any cancer	6.0	7.2	5.5	5.6	4.4
Mild to severe liver disease	4.8	4.7	4.8	4.8	4.9
Myocardial infarction	2.3	3.1	2.2	1.5	1.6
Rheumatoid arthritis and other inflammatory polyarthropathies	1.6	1.8	1.3	1.5	1.5

*SD, standard deviation*.

### PCs

**Figure 1:**
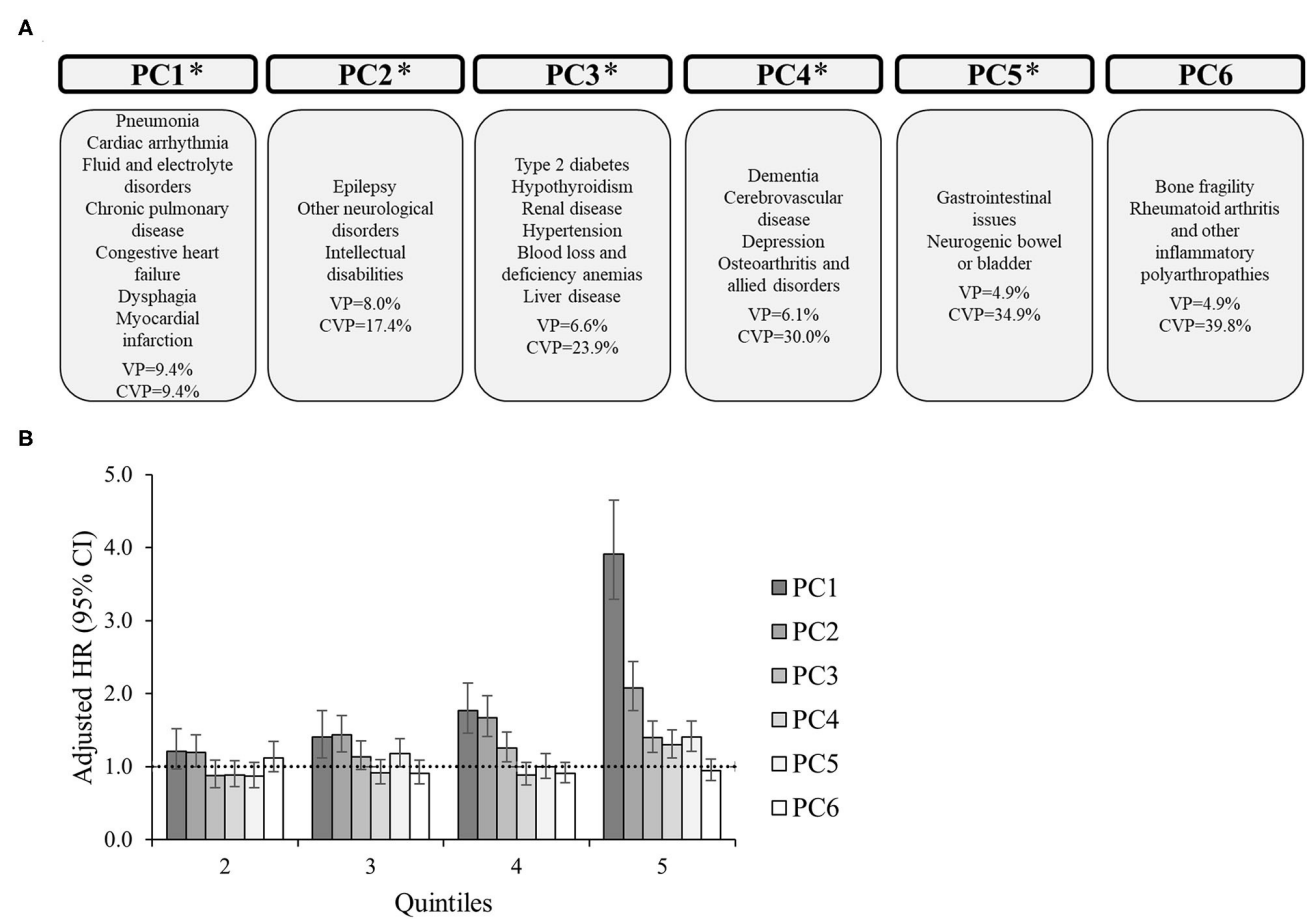
Principal component analysis among 16,728 adults with cerebral palsy. **(A)** Principal components (PC) represent the comorbidity combinations in order of the amount of variance percent (VP) explained and the cumulative VP (CVP) explained. *P < 0.05 for the association of that PC with 2-year mortality. **(B)** The association between each PC with 2-year mortality after PC scores were transformed into quintiles to facilitate comparisons across PCs (reference: lowest quintile). The bars represent the hazard ratio (HR) and the vertical lines represent the 95% confidence interval (CI) for 2-year mortality after adjusting for all PCs, age, sex, race, and U.S. region of residence. The dotted horizontal line represents a HR of 1.0. If the 95% CI crosses the dotted line, the association is not statistically significant at *P* = 0.05.

For the entire group, there were 6 PCs (Figure 1) that explained 39.8% of the total variance. PC1 generally includes cardiorespiratory diseases, as well as fluid/electrolyte disorders and dysphagia, explaining 9.4% of the variance. PC2 includes neurological disorders, explaining 8.0% of the variance (cumulative, 17.4%). PC3 generally includes metabolic disorders, specifically type 2 diabetes, hypothyroidism, renal disease, hypertension, anemias, and liver disease, explaining 6.6% of the variance (cumulative, 23.9%). PC4 generally includes brain-related disorders, specifically dementia, cerebrovascular disease, and depression, as well as osteoarthritis, explaining 6.1% of the variance (cumulative, 30.0%). PC5 includes gastrointestinal issues and neurogenic bowel or bladder, explaining 4.9% of the variance (cumulative, 34.9%). Finally, PC6 includes orthopedic-related disorders, specifically bone fragility and rheumatoid arthritis and other inflammatory polyarthropathies, explaining 4.9% of the variance (cumulative, 39.8%).

**Figure 2:**
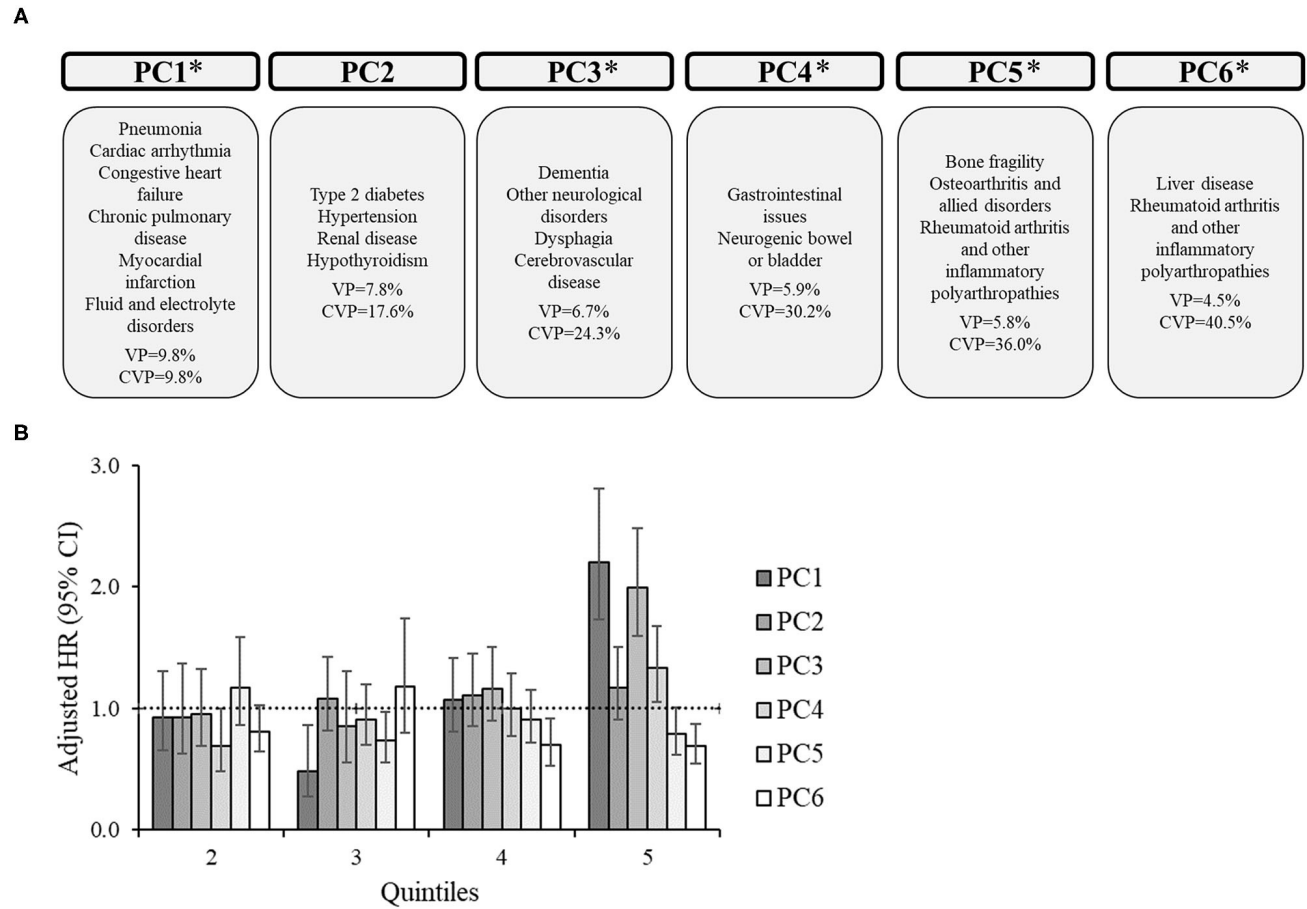
Principal component analysis among 7,542 adults with cerebral palsy without co-occurring epilepsy and intellectual disabilities. **(A)** Principal components (PC) represent the comorbidity combinations in order of the amount of variance percent (VP) explained and the cumulative VP (CVP) explained. *P < 0.05 for the association of that PC with 2-year mortality. **(B)** The association between each PC with 2-year mortality after PC scores were transformed into quintiles to facilitate comparisons across PCs (reference: lowest quintile). The bars represent the hazard ratio (HR) and the vertical lines represent the 95% confidence interval (CI) for 2-year mortality after adjusting for all PCs, age, sex, race, and U.S. region of residence. The dotted horizontal line represents a HR of 1.0. If the 95% CI crosses the dotted line, the association is not statistically significant at *P* = 0.05.

**Figure 3:**
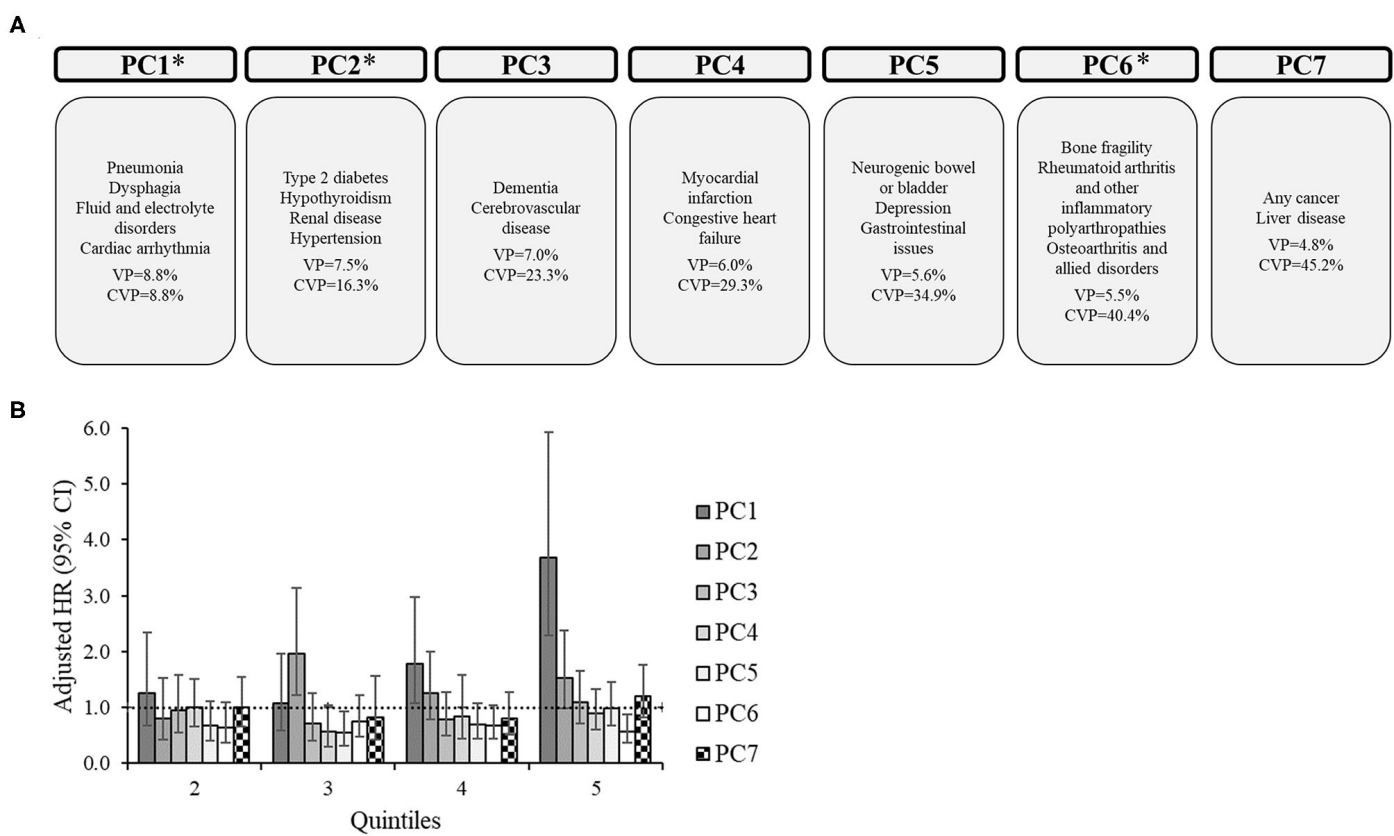
Principal component analysis among 2,607 adults with cerebral palsy with co-occurring epilepsy, but without co-occurring intellectual disabilities. **(A)** Principal components (PC) represent the comorbidity combinations in order of the amount of variance percent (VP) explained and the cumulative VP (CVP) explained. *P < 0.05 for the association of that PC with 2-year mortality. **(B)** The association between each PC with 2-year mortality after PC scores were transformed into quintiles to facilitate comparisons across PCs (reference: lowest quintile). The bars represent the hazard ratio (HR) and the vertical lines represent the 95% confidence interval (CI) for 2-year mortality after adjusting for all PCs, age, sex, race, and U.S. region of residence. The dotted horizontal line represents a HR of 1.0. If the 95% CI crosses the dotted line, the association is not statistically significant at *P* = 0.05.

For the subgroups, there were 6 PCs for CP only (Figure 2), 7 PCs for CP+EP (Figure 3), 7 PCs for CP+ID (Supplementary Figure 1), and 7 PCs for CP+EP+ID (Supplementary Figure 2), that explained 40.5, 45.2, 43.9, and 44.2%, respectively, of the total variance. Notably, all subgroups had a similar PC1 as the entire group (explaining 8.8–10.8% of the variance), generally including cardiorespiratory diseases, and fluid/electrolyte disorders, and dysphagia. Renal disease, type 2 diabetes, hypertension, and hypothyroidism were included in PC2 for CP only and CP+EP, whereas PC2 for CP+ID included type 2 diabetes, hypertension, and blood loss and deficiency anemias, and PC2 for CP+EP+ID included type 2 diabetes, hypertension, and hypothyroidism. Dementia and cerebrovascular disease were together in PC3, among other morbidities, for each subgroup.

### Association Between PCs With Mortality

During the follow-up period for a mean (SD) of 696 (126) days (median, 730 days), 1,486 (8.9%) died from the entire group, 27 (0.1%) were right-censored due to loss of follow-up, and 15,215 (91.0%) were right-censored due to the end of the study period. The follow-up time was similar for the subgroups. In total, 602 (8.0%) died from CP only, 203 (7.8%) died from CP+EP, 260 (9.4%) died from CP+ID, and 421 (11.1%) died from CP+EP+ID.

The adjusted HR of 2-year mortality after including all PCs in the model as quintiles (reference: lowest quintile) is presented in Figures 1–3 and Supplementary Figures 1, 2 alongside the PC comorbidity grouping. For the entire group (Figure 1), PC1-PC5 were significantly associated with an elevated HR of mortality, especially PC1. For example, the adjusted HR (95% CI) for the 5th quintile for PC1 (reference: 1st quintile) was 3.91 (3.29–4.65), which was higher than the HRs of the 5th quintile from PC2-PC5 (HR range, 1.21–2.08, all *P* < 0.05).

PC1 was significantly associated with an elevated HR of mortality for each subgroup, which included similar comorbidities (e.g., cardiorespiratory diseases) across subgroups. Additionally, PC1 had the highest HR for the 5th quintile compared to other PCs for each subgroup. There were unique associations between PCs with mortality for each subgroup.

## Discussion

This study identified the most common comorbidity combinations among adults with cerebral palsy with and without co-occurring epilepsy and/or intellectual disabilities, advancing the knowledge of what multimorbidity looks like for this population, and additionally, what it may mean for survival. These findings inform on the potential underlying shared set of etiologies or characteristics among these combinations of comorbidities unique to adults with cerebral palsy. Some comorbidity combinations were more obvious given what is known in the field, such as dysphagia and pneumonia in PC1. Some comorbidity combinations were less obvious, and may inform on new shared pathophysiologic pathways among comorbidities not previously regarded as established knowledge in the field, such as dysphagia and myocardial infarction, or the comorbidities present in PC3 for the entire sample.

It is important to note that the most common comorbidity combinations in the entire sample explained ~40% of the total variance. The total variance explained for defining multimorbidity profiles in this study is between that previously reported from other studies among adults without cerebral palsy (15–65%) (24–26). The difference between studies may be due to differences in the number of morbidities examined and criteria used, among other factors. The current study examined an extensive list of clinically relevant morbidities (15) and applied more stringent criteria (e.g., factor loading) to minimize the possibility of type I error. Nevertheless, the 40% of total variance explained could suggest significant heterogeneity of multimorbidity profiles for adults with cerebral palsy, which is likely confirming what many in the field have suspected. Further, the heterogeneity may be in part due to the different multimorbidity profiles associated with co-occurring intellectual disabilities and/or epilepsy. When these subgroups were stratified, their most common comorbidity combinations explained slightly more of the total variance (~45%) compared to the entire sample (~40%). Indeed, while many of the same comorbidities were present in PCs across subgroups, unique comorbidity combinations emerged for each of the subgroups, providing more personalized information for the broader adult population with cerebral palsy.

As expected, the PCs had general themes, with PC1 including several cardiorespiratory diseases along with dysphagia and fluid/electrolyte disorders. PC1 was also the deadliest for the entire sample and all subgroups. This finding may be in part driven by those with more severe forms of cerebral palsy, as severity of cerebral palsy is associated with a greater risk of respiratory diseases, dysphagia, and premature mortality, as well as mal-nourishment contributing to fluid/electrolyte disorders (27, 28). The quintile pattern of the association of PC1 with mortality suggests that, after full adjustments, those that have a moderate profile of PC1 have an increased rate of mortality (e.g., entire sample 3rd quintile HR = 1.41), but those with a more exemplary profile of PC1 have a substantially increased rate of mortality (e.g., entire sample 5th quintile, HR = 3.91). This could suggest that cardiorespiratory diseases are deadly for adults with cerebral palsy, which is consistent with previous studies (29), but that the combination with dysphagia and possibly fluid/electrolyte disorders are especially deadly, making this finding unique to cerebral palsy. Importantly, dysphagia and fluid/electrolyte disorders can both be clinically managed, but are likely overlooked due to lack of clinicians familiar with these problems when treating adults with cerebral palsy, thus exacerbating a preventable health disparity that may be associated with an excess risk of mortality.

Notably, this study identified that in the entire sample and each subgroup, dementia and cerebrovascular disease were together in a PC. Further, for the entire group and subgroups with ID, depression was included in the PC with dementia and cerebrovascular disease. There is growing interest in the neurological field in examining dementia risk for adults with cerebral palsy (6, 8). However, it can be challenging to identify dementia risk using the currently available methods. Clinically, this study may provide an indirect path for assessment of dementia, by considering that those with cerebrovascular disease (and depression) may also be likely to have dementia; although, this is speculation and more work in this area is needed (8). Biologically, this novel finding may suggest an underlying etiology or shared characteristics among dementia, cerebrovascular disease, and depression in cerebral palsy. Early life stressors are known to alter epigenetic modifications leading to later-life brain-related abnormalities, cardiometabolic disease, and mental health disorders, providing a biologically embedded link between childhood adversity and adult health (30–32). Crowgey et al. (33) found distinct altered epigenetic patterns in peripheral blood cells in adolescents with vs. without cerebral palsy. More research is certainly needed into the role of early life stressors (e.g., damage to brain causing cerebral palsy, psychosocial stressors) as a biologically plausible link to poor health in adults with cerebral palsy.

Interestingly, for the entire sample and for CP+EP+ID, but not the other subgroups, osteoarthritis loaded onto the factor containing cerebrovascular disease, dementia, and depression. While often regarded as an orthopedic or peripheral pain condition, osteoarthritis has been shown to modify brain activity over time, and increase risk of dementia, stroke, and mental health disorders through several pathways, and can also re-direct pain experience and symptoms among centralized brain networks (34–37). This could suggest that osteoarthritis can act as both a peripheral and central nervous system condition, which may uniquely manifest and integrate with other health problems for people with cerebral palsy. However, more work is needed to understand these pathways and why this was more relevant to the subgroup with CP+EP+ID.

This study also identified that diabetes and hypertension were present in PC2 in all subgroups, where renal disease was additionally combined in CP only, CP+EP, and the entire sample (for PC3), indicating that these comorbidity combinations are relatively common among adults with cerebral palsy (based on the position of the PC). This is consistent with previous studies that have shown that these 3 morbidities often co-occur in the general population (16, 24) and adults with cerebral palsy (38). Further, diabetes and hypertension have been found to be robust risk factors for developing advanced stages of chronic kidney disease among adults with cerebral palsy (39). In the current study, the PC that was defined in part by diabetes, renal disease, and hypertension for the entire sample and subgroups was associated with an elevated rate of mortality. The duality of these comorbidity combinations presenting as (1) relatively common and (2) deadly could provide a basis for clinical monitoring and justification to insurance providers for additional testing.

For the entire sample, PC6, defined by bone fragility and rheumatoid arthritis, was not associated with mortality. This may shed some light on a newly forming concept in the field that bone fragility is implicated in the pathogenesis of unhealthful aging for adults with cerebral palsy. A recent epidemiologic study have found that sustaining a fragility fracture is a robust risk factor for premature mortality for adults with cerebral palsy, even after accounting for demographics, cardiorespiratory diseases, diabetes, cancer, and kidney disease (40). Additional epidemiologic studies have also found that fragility fractures are a robust risk factor for incidence of several cardiorespiratory diseases for adults with cerebral palsy (41–44). Fractures therefore may have an “up-stream” effect on mortality risk by orchestrating a cascade of events (e.g., altered biology, loss of function) that contributes in part to the increased risk of cardiorespiratory diseases, which in turn drives premature mortality, as cardiorespiratory diseases are among the primary causes of premature mortality for adults with cerebral palsy (29). Since this study identified comorbidities in a cross-sectional manner, the temporal sequence of fracture longitudinally leading to cardiorespiratory disease would not be captured, helping to explain why these conditions were not present together in a PC.

Limitations of this study based on the use of administrative claims data for adults with cerebral palsy has been reported previously (10, 38, 45–47). It is worth re-mentioning the potential issue for healthcare providers in accurately detecting and diagnosing mental health disorders and dementia, especially for individuals that have intellectual disabilities or trouble communicating. As a result, mental health disorders and dementia may be under-represented in this study. It is also worth re-mentioning that many cerebral palsy-related characteristics are not available in administrative claims data, such as severity of cerebral palsy. In some research contexts studying multimorbidity, this is not a major limitation, as the number of comorbidities is associated with severity of cerebral palsy (11, 12). However, the accumulation and combinations of comorbidities may differ by severity of cerebral palsy due to the differences in body development, biology, and medical needs. Future research is needed to identify comorbidity combinations by severity of cerebral palsy. Along these lines, claims data do not contain information about nutrition or body composition measures, such as body mass, waist circumference, or body mass index, preventing the ability to examine the influence by body composition. Claims data do not contain information as to the cause of death, so it is unknown what that actual cause of deaths were in this study. This study was also focused on morbidities and not medications. The number of and composition of medications may promote drug interactions creating new medical problems or exacerbating current medical problems. Future studies are needed to understand the role of medications along with morbidities in the context of aging with cerebral palsy. Finally, as there is a lack of general consensus on best methodologies for PCA using dichotomous variables to define multimorbidity profiles, this study opted for more stringent criteria to provide a simpler and more robust conclusion. However, the cost can be lack of placement of comorbidities in certain combinations, providing an incomplete assessment of comorbidities that may further inform on the underlying shared pathophysiology.

This study provides empirical evidence of the most common comorbidity combinations among adults with cerebral palsy with and without co-occurring epilepsy and/or intellectual disabilities, thus enhancing interpretations for the broader adult population with cerebral palsy. This study also observed that many of the comorbidity combinations were associated with an elevated mortality rate, but to a differing degree. Taken together, study findings provide a comprehensive qualitative assessment of multimorbidity, informing on the potential underlying shared etiologies or characteristics of comorbidities unique to adults with cerebral palsy.

## Data Availability Statement

Publicly available datasets were analyzed in this study. This data can be found here: the data analyzed in this study was obtained from the Centers for Medicare & Medicaid Services, the following licenses/restrictions apply: The dataset analyzed in this study may be accessed through a contractual agreement with the Centers for Medicare & Medicaid Services following the payment of an administrative fee. Information about these datasets and requests for dataset access can be found at: https://www.cms.gov/Research-Statistics-Data-and-Systems/Research-Statistics-Data-and-Systems.

## Author Contributions

DW designed the study, analyzed the data, and wrote the first draft of the manuscript. MS and EH edited the manuscript. All authors conceptualized the study, approve the final version of this manuscript, and agree to be accountable for the content of the work.

## Funding

This work was supported by the University of Michigan Office of Health Equity and Inclusion Diversity Fund (DW). The funding source had no role in the design and conduct of the study; collection, management, analysis, and interpretation of the data; preparation, review, or approval of the manuscript; and decision to submit the manuscript for publication.

## Conflict of Interest

The authors declare that the research was conducted in the absence of any commercial or financial relationships that could be construed as a potential conflict of interest.

## Publisher's Note

All claims expressed in this article are solely those of the authors and do not necessarily represent those of their affiliated organizations, or those of the publisher, the editors and the reviewers. Any product that may be evaluated in this article, or claim that may be made by its manufacturer, is not guaranteed or endorsed by the publisher.

## Supplementary Material

Supplementary Figure 1 Principal component analysis among 2,781 adults with cerebral palsy with co-occurring intellectual disabilities, but without co-occurring epilepsy. **(A)** Principal components (PC) represent the comorbidity combinations in order of the amount of variance percent (VP) explained and the cumulative VP (CVP) explained. **P* < 0.05 for the association of that PC with 2-year mortality. **(B)** The association between each PC with 2-year mortality after PC scores were transformed into quintiles to facilitate comparisons across PCs (reference: lowest quintile). The bars represent the hazard ratio (HR) and the vertical lines represent the 95% confidence interval (CI) for 2-year mortality after adjusting for all PCs, age, sex, race, and U.S. region of residence. The dotted horizontal line represents a HR of 1.0. If the 95% CI crosses the dotted line, the association is not statistically significant at *P* = 0.05.

Supplementary Figure 2 Principal component analysis among 3,798 adults with cerebral palsy with co-occurring epilepsy and intellectual disabilities. **(A)** Principal components (PC) represent the comorbidity combinations in order of the amount of variance percent (VP) explained and the cumulative VP (CVP) explained. **P* < 0.05 for the association of that PC with 2-year mortality. **(B)** The association between each PC with 2-year mortality after PC scores were transformed into quintiles to facilitate comparisons across PCs (reference: lowest quintile). The bars represent the hazard ratio (HR) and the vertical lines represent the 95% confidence interval (CI) for 2-year mortality after adjusting for all PCs, age, sex, race, and U.S. region of residence. The dotted horizontal line represents a HR of 1.0. If the 95% CI crosses the dotted line, the association is not statistically significant at *P* = 0.05.
