# Corrigendum: Shared Physiologic Pathways Among Comorbidities for Adults With Cerebral Palsy

**DOI:** 10.3389/fneur.2021.830052

**Published:** 2022-01-18

**Authors:** Daniel G. Whitney, Mary Schmidt, Edward A. Hurvitz

**Affiliations:** ^1^Department of Physical Medicine and Rehabilitation, University of Michigan, Ann Arbor, MI, United States; ^2^Institute for Healthcare Policy and Innovation, University of Michigan, Ann Arbor, MI, United States

**Keywords:** cerebral palsy, comorbidities, mortality, multimorbidity, clinical epidemiology

In the original article, there was an error in the reporting of one of the statistical approaches in the Methods section. The original reporting stated that the principal component analysis model used a tetrachoric correlation matrix, but the model did not use this matrix. Specifically, the error was in the section *Methods, Statistical Analysis, Paragraph 1*, which stated: “The PCA models used a tetrachoric correlation matrix due to the dichotomous nature of the variables, and a varimax rotation was used to facilitate interpretation of the loading factors.”

A correction has been made to *Methods, Statistical Analysis, Paragraph 1*. The corrected paragraph is shown below.

PCA is a multivariable data reduction technique that operates in a highly correlated environment to identify inter-correlated variables. In the context of multimorbidity, PCA allows for meaningful analysis and interpretation of comorbidity data by reducing the number of comorbidities into a few inter-correlated combinations. Each comorbidity combination corresponds to a specific principal component (PC) (21, 22), which is independent of other PCs. To derive the PCs, the models included each of the WCI comorbidities (25 for the entire group; 23 for the subgroups). The PCA models used a varimax rotation to facilitate interpretation of the loading factors. Loading factors are derived from the correlation matrix and provides a numerical interpretation of the PCs. Comorbidities with a loading factor of ≥|0.40| were included for interpretation, which has been suggested previously (23). PCs with eigenvalues of ≥1.00 were retained and analyzed, as this is common practice for PCA (24).

The authors apologize for this error and state that this does not change the scientific conclusions of the article in any way. The original article has been updated.

## Publisher's Note

All claims expressed in this article are solely those of the authors and do not necessarily represent those of their affiliated organizations, or those of the publisher, the editors and the reviewers. Any product that may be evaluated in this article, or claim that may be made by its manufacturer, is not guaranteed or endorsed by the publisher.

