# Supplementary figures and images for: Shared Physiologic Pathways Among Comorbidities for Adults With Cerebral Palsy
Source: Front Neurol. 2021 Oct 4;12:742179. doi: 10.3389/fneur.2021.742179 (PMC8520916; doi:10.3389/fneur.2021.742179)

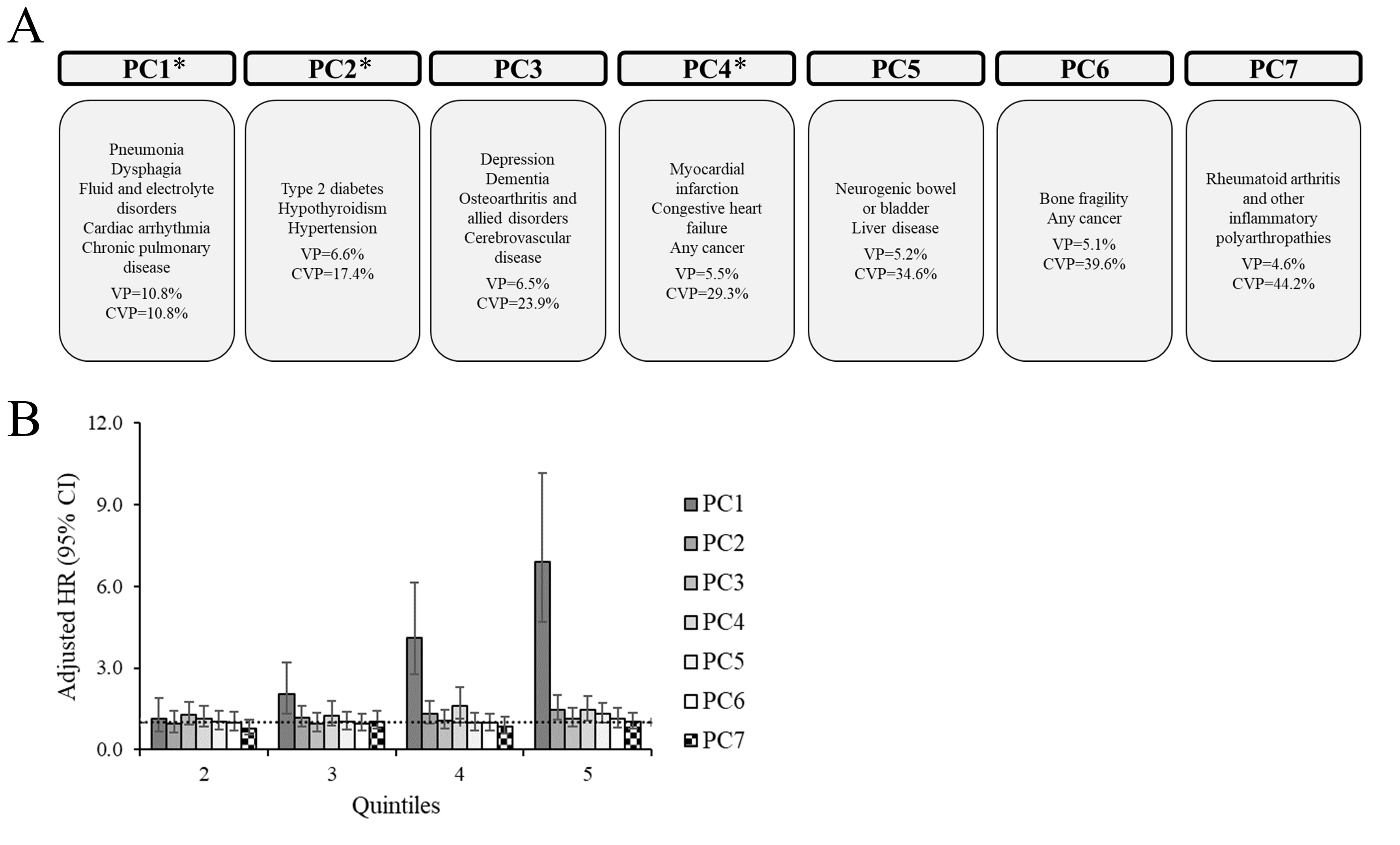

Supplement: Supplementary Figure 1 — Principal component analysis among 2,781 adults with cerebral palsy with co-occurring intellectual disabilities, but without co-occurring epilepsy. (A) Principal components (PC) represent the comorbidity combinations in order of the amount of variance percent (VP) explained and the cumulative VP (CVP) explained. *P < 0.05 for the association of that PC with 2-year mortality. (B) The association between each PC with 2-year mortality after PC scores were transformed into quintiles to facilitate comparisons across PCs (reference: lowest quintile). The bars represent the hazard ratio (HR) and the vertical lines represent the 95% confidence interval (CI) for 2-year mortality after adjusting for all PCs, age, sex, race, and U.S. region of residence. The dotted horizontal line represents a HR of 1.0. If the 95% CI crosses the dotted line, the association is not statistically significant at P = 0.05. [file Image_1.TIF]

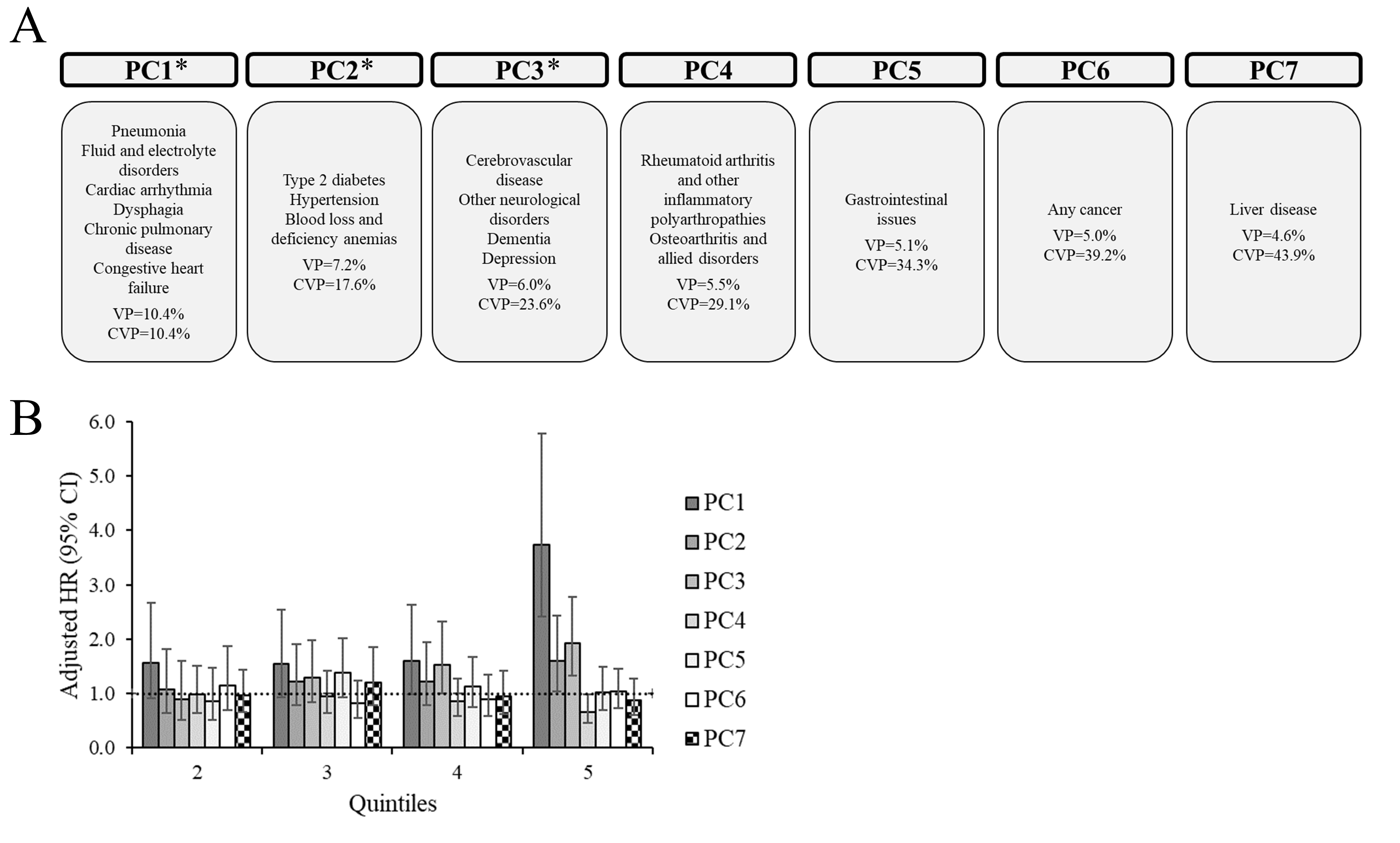

Supplement: Supplementary Figure 2 — Principal component analysis among 3,798 adults with cerebral palsy with co-occurring epilepsy and intellectual disabilities. (A) Principal components (PC) represent the comorbidity combinations in order of the amount of variance percent (VP) explained and the cumulative VP (CVP) explained. *P < 0.05 for the association of that PC with 2-year mortality. (B) The association between each PC with 2-year mortality after PC scores were transformed into quintiles to facilitate comparisons across PCs (reference: lowest quintile). The bars represent the hazard ratio (HR) and the vertical lines represent the 95% confidence interval (CI) for 2-year mortality after adjusting for all PCs, age, sex, race, and U.S. region of residence. The dotted horizontal line represents a HR of 1.0. If the 95% CI crosses the dotted line, the association is not statistically significant at P = 0.05. [file Image_2.TIF]
